# Prevalence estimation of essential tremor in Hungary between 2010 and 2020 based on the National Health Insurance Fund Database

**DOI:** 10.1038/s41598-025-13145-6

**Published:** 2025-08-05

**Authors:** Andrea Kinga Papp, Ádám József Berki, Péter Vinnai, András Ajtay, Dániel Bereczki, Loránd Erőss, Gertrúd Tamás

**Affiliations:** 1 Department of Neurology, Semmelweis University, Budapest, Hungary; 2 HUN-REN SU Neuroepidemiological Research Group, Budapest, Hungary; 3 Department of Neurosurgery and Neurointervention, Semmelweis University, Budapest, Hungary

**Keywords:** Epidemiology, Essential tremor, Hungary, Prevalence, Therapy, Tremor, Diseases, Health care, Neurology

## Abstract

The published prevalence of essential tremor is variable worldwide and lacking in Central Europe. We aimed to estimate its prevalence in Hungary and to explore the locally applied therapeutic approaches. We collected data from the National Health Insurance Fund database and pharmacy database registered between 2010 and 2020. Matching the specified codes of the International Classification of Diseases and the individually tailored combination of interventions, we attempted to exclude Parkinson’s disease and other tremor evoking conditions. We estimated the period prevalence of essential tremor age standardized to the European Standard Population at 378–388/100,000. After excluding patients with possible Parkinsonian syndromes, we found that 36.4% of patients with tremor did not take any medication during the study period. Most of the rest used alprazolam followed by propranolol for the longest period of time; the alprazolam and propranolol combination was the most preferred. Deep brain stimulation and ablative surgery were chosen for less than 0.5% of the patients. Our strict methods probably underestimate the essential tremor prevalence in Hungary, which, however, does not differ considerably from international results. Given the limitations of medication therapy, expanding and improving neurosurgical interventions may help improve the quality of life of patients with essential tremor.

## Introduction

Essential tremor (ET) is an isolated tremor syndrome of bilateral upper limb postural or kinetic tremor, sometimes accompanied by head tremor or tremor in other body parts^1^. Its differential diagnosis is often a challenge. Essential tremor associated with soft neurological signs, such as impaired tandem gait, mild memory impairment, or subtle dystonic posturing, is labeled essential tremor plus (ET plus) syndrome. Tremor appears in several neurodegenerative, mitochondrial, infectious, endocrine, or metabolic disorders, or it is evoked by some frequently administered drugs or toxins^1^. In the early stages of Parkinson’s disease, essential tremor is the most common misdiagnosis^2^.

Although essential tremor is a benign disorder, it can substantially decrease the quality of life^3^ as many patients experience a regular and consistent worsening in their condition^4^. Furthermore, many patients feel hopeless in coping with their illness; demoralization was found to be nearly as frequent in essential tremor as in chronic or terminal diseases, such as in cancer, coronary heart disease, or Parkinson’s disease^5 ^highlighting the need to discover local therapeutic habits in this patient population.

In evidence-based recommendations, propranolol, primidone, and topiramate (in a daily dose higher than 200 mg) were categorized as clinically useful, while alprazolam as possibly useful in ET^6,7^. Propranolol and primidone are frequently administered in combination because they synergize^8^. Botulinum toxin A may have a beneficial effect in selected cases^9^. Unilateral stimulation of the ventral intermedial nucleus of the thalamus, unilateral focused ultrasound thalamotomy, as well as radiofrequency thalamotomy, are possibly effective in essential tremor with acceptable risks, according to the latest guideline^6^. Bilateral neurosurgical procedures are still under investigation for creating evidence-based recommendations on their use; however, according to expert opinion, similarly to unilateral surgery, they offer better outcomes (with an estimated improvement of around 80%) than medication therapies^7^.

Essential tremor is considered to be one of the most common movement disorders in adults; it is up to 4–5 times more common than Parkinson’s disease in previously published studies^10–12^. Its prevalence after the age of 65 years is close to that of Alzheimer’s disease^13^. There is remarkably less epidemiological data from Europe than from the Asian continent^14^. The prevalence of essential tremor has been studied in only a few European countries (Finland^15^,  Italy^16,17^, Spain^18^,  Faroe Islands^19^) and not in the Central European regions; the prevalence of ET in these countries ranges from 0.4%^16^ to 1.4% in the general population^20^ and up to 8.6% in people over 65 years^18^. Some population-based studies investigate only small geographical territories^19,21^.

In this study, we aimed to calculate the ET prevalence in Hungary, despite the difficulties of its differential diagnosis; and to analyze the use of available therapeutic approaches recommended by international guidelines. We wanted to explore the necessary public health resources and the nationally unmet challenges for preparing public health actions^14^ to optimize the care of ET.

## Results

### Grouping the multiple causes of tremor

**Fig. 1:**
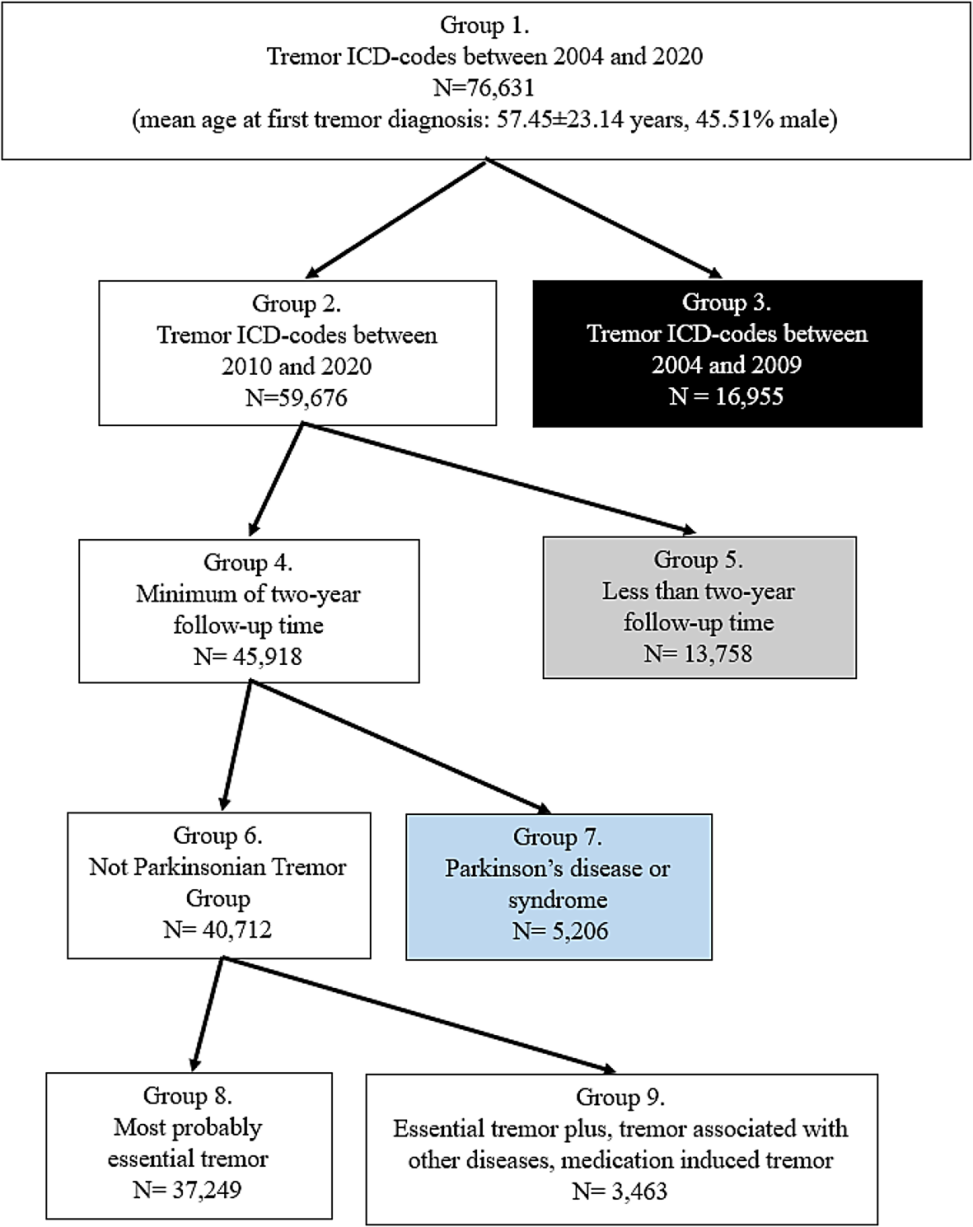
** Flowchart of the selection process**. The flowchart diagram shows the steps of selection from our initial database to the final established number of patients with essential tremor. We excluded the patients represented in the colored boxes from the study. Patients represented in the black box had no pharmacy records (Group 3); in the gray box, they had less than two years of follow-up time (Group 5). Patients depicted in the blue box were considered to have Parkinson’s disease/syndrome (Group 7). The remaining Not Parkinsonian Tremor Group contained data from 40,712 patients (Group 6). After ruling out people with possible Essential tremor plus syndromes and other tremor-eliciting conditions and treatments (Group 9), the estimated number of patients with essential tremor was 37,249 (Group 8).

Figure 1 depicts the flowchart of the patient selection. We found 76,631 patients (Group 1) who received any of the four specified tremor ICD-codes between 2004 and 2020 (the mean age at the first tremor diagnosis was 57.45 ± 23.14 years, 45.51% male). Due to the limited access to the pharmacy database of medication refills (2010–2020), we excluded 16,955 patients (Group 3) who only received tremor codes between 2004 and 2009. There were 7,317 patients included in the study who not only had their first tremor diagnosis between 2004 and 2009, but also had tremor ICD-codes between 2010 and 2020. Finally, we analyzed 59,676 patients (Group 2) who received tremor codes during our actual study period between 2010 and 2020.

We excluded 13,758 patients (Group 5) from the analysis for lack of a sufficient two years’ follow-up period. There were 45,918 patients (Group 4) with a minimum of two years of observation time; they were further investigated regarding medication use and differential diagnosis. A group of 5,206 patients (Group 7) used antiparkinsonian medications in at least 80% of the study period, starting after the initial 2 years following their first tremor diagnosis; this group of patients was considered to have Parkinson’s disease/syndrome and thus were separated from the Not Parkinsonian Tremor Group (Group 6).

In the Not Parkinsonian Tremor Group, we explored the likelihood of each alternative diagnosis outside Parkinsonian syndromes and additional signs associated with tremor, which was less than 1% (Table 1). In the Not Parkinsonian Tremor Group, the ratio of patients taking amiodarone, valproate, and l-thyroxin varied between 1 and 3%; the ratio was lower for lithium (Table 1).

**Table 1:** Tremor-eliciting conditions and treatments in Group 6.

	Number (percentage) of patients in Group 6
Tremor-eliciting conditions	
Alcohol abuse	265 (0.65%)
Hyperthyroidism	242 (0.59%)
Dystonia	164 (0.4%)
Multiple sclerosis	137 (0.34%)
Blepharospasm	119 (0.29%)
Torticollis	30 (0.07%)
Wilson’s disease	14 (0.03%)
Tremor-eliciting treatments	
Amiodarone	1285 (3.15%)
Valproate	849 (2.08%)
l-Thyroxin	403 (1%)
Lithium	186 (0.45%)

We found tremor-eliciting conditions in less than 3% of the patients; none had a frequency higher than 1%. Among the medications that might evoke enhanced physiological tremor, amiodarone was most frequently found, followed by valproate.

Overall, 3,463 patients (Group 9) had at least one alternative or additional diagnosis/sign and/or had consumed at least one of the potentially tremor-inducing medications. In the case of 37,249 patients (Group 8), these methods excluded alternative causes of tremor, so they were considered to have most probably essential tremor.

### Therapeutic approaches

**Fig. 2:**
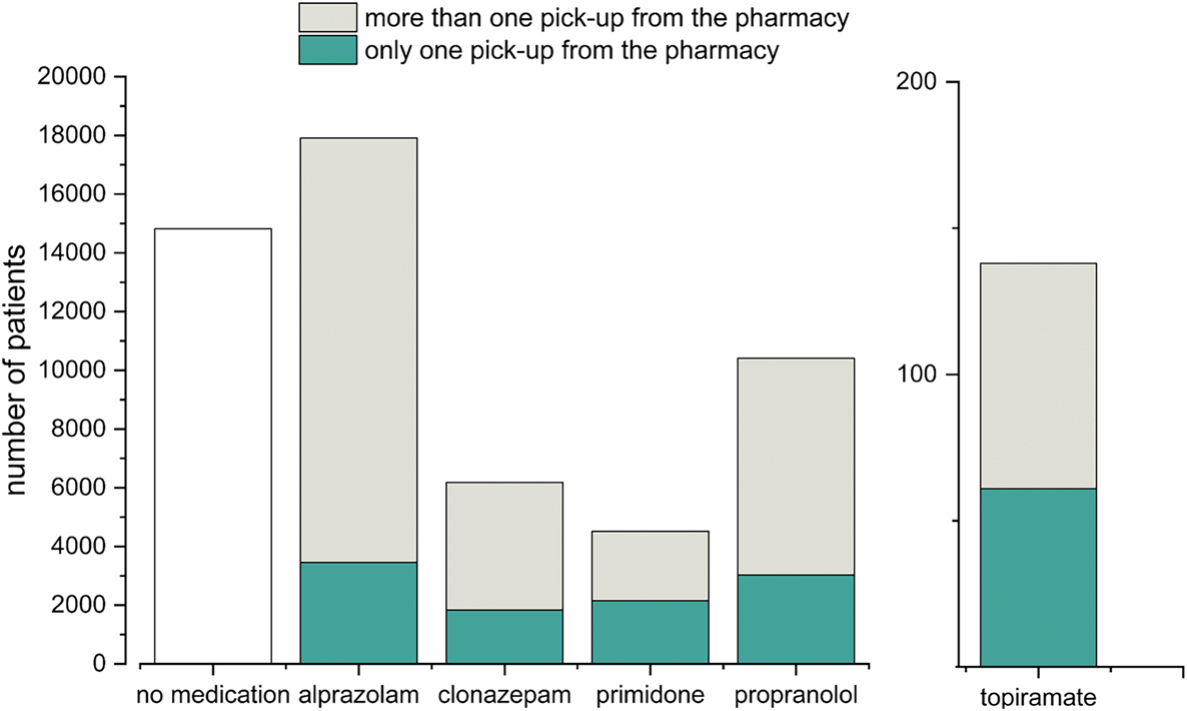
Customs of medication refills in the Not Parkinsonian Tremor Group (Group 6). During our investigation period, 14,825 patients did not try any studied medication. Most of those being on pharmacotherapy picked up the prescription of alprazolam one or more times. The ratio of those who refilled the respective medication more than once compared to those who asked for it only once at the pharmacy, as a metric of adherence, was the highest for alprazolam. Only 138 patients used topiramate.

**Fig. 3:**
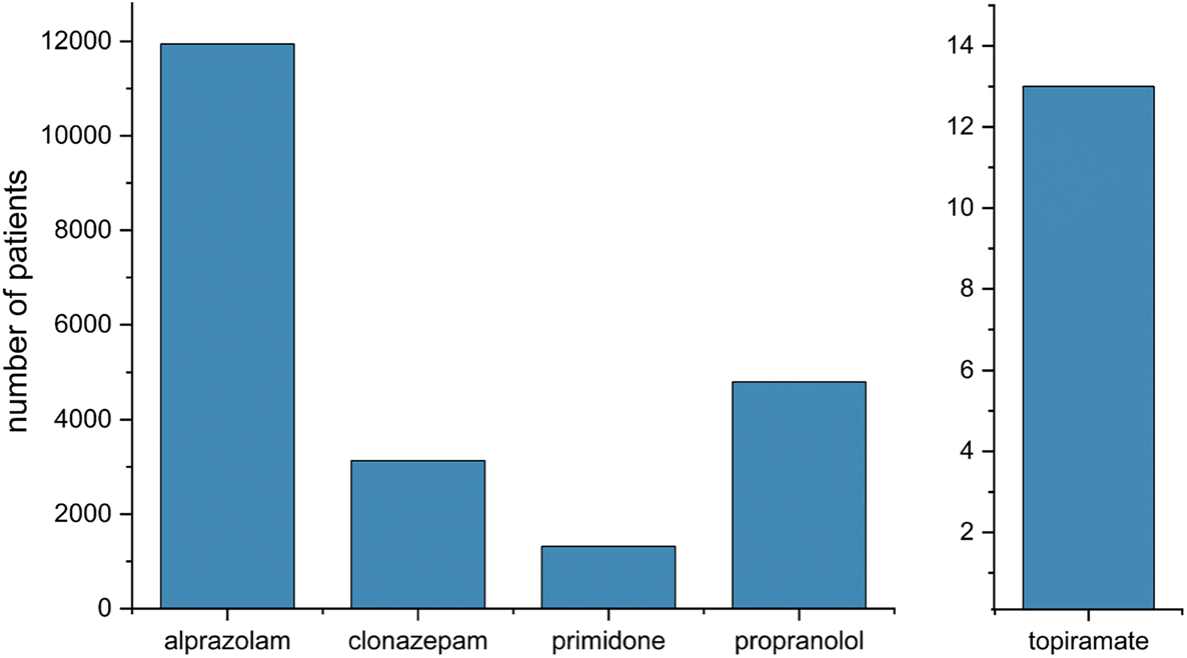
The number of patients in Group 6 using the represented antitremor drug for the most extended period of time. Each column represents the number of patients who refilled a particular medication most prolongedly out of the five studied ones during our observation period. Most patients opted for alprazolam for the most extended period of time.

**Fig. 4:**
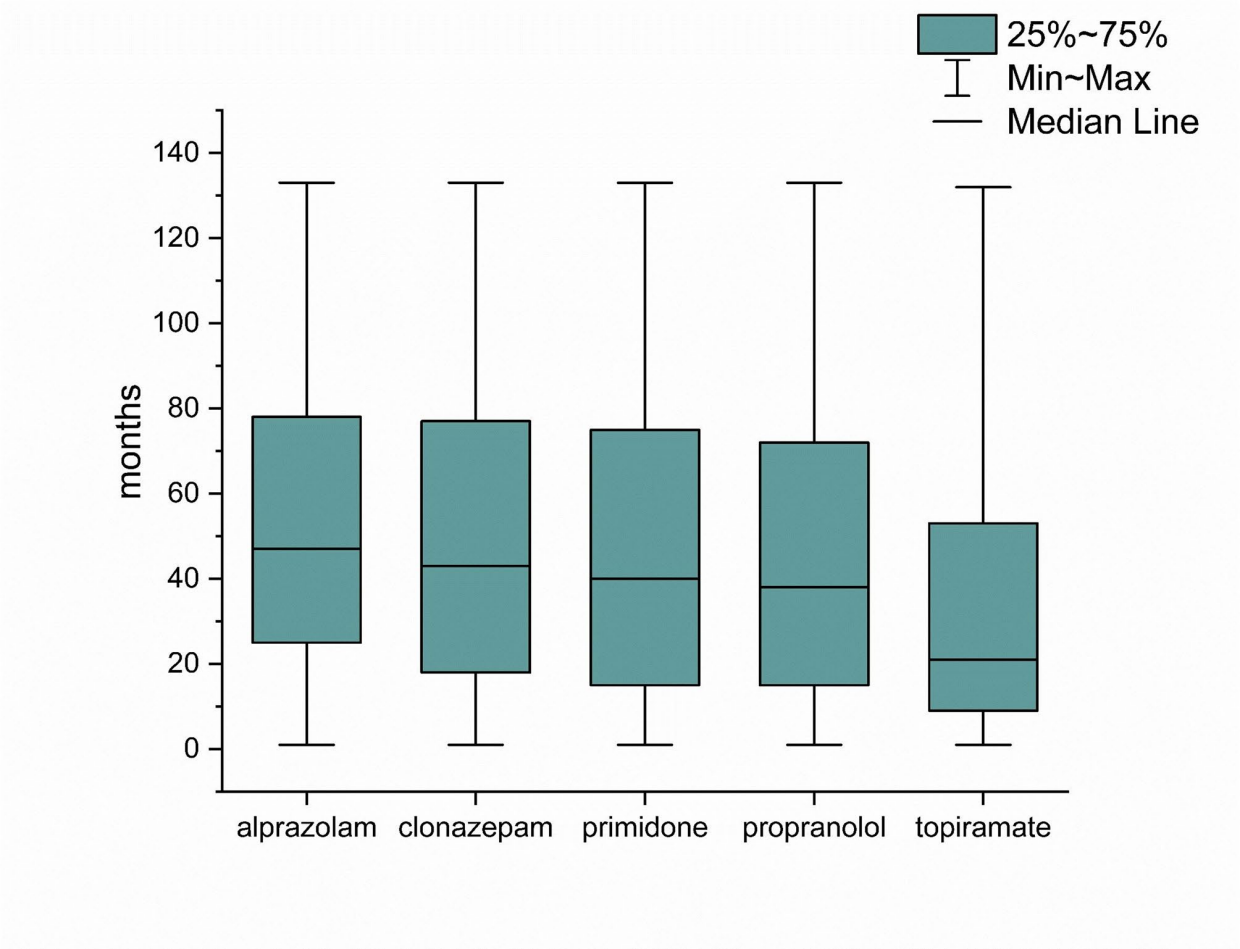
The lengths of the medication use in Group 6. The plot shows the time period in months when the patients refilled the listed drugs from the pharmacy. The duration of medication uses significantly differed (p ***<*** 0.0001, Kruskal-Wallis test), being the longest for alprazolam and the shortest for topiramate. Post hoc Dunn’s test showed that the difference was significant between each medication (p ***<*** 0.05), except for the comparison between propranolol and primidone (*p* > 0.05; NS).

Regarding pharmacological approaches, we found that in the Not Parkinsonian Tremor Group (Group 6), 14,825 patients (36.41%) never tried any of the five studied medications (propranolol, primidone, topiramate, alprazolam, and clonazepam). A group of 7,459 patients (18.32%) consumed any of these medicines in less than 20% of the follow-up period. Based on this data, 22,284 patients (54.73%) did not consume any specific medication in at least 80% of the investigated time interval. Alprazolam was dispensed to 44% of those patients who were taking any of the studied medications. (Fig. 2). Furthermore, Table 2 shows that among medications taken at least once during the study period, the highest percentage of patients preferred benzodiazepines. Among the five studied antitremor medications, 11,943 patients utilized alprazolam for the longest period of time (Fig. 3). The duration of medication use, when the patients refilled the listed drugs from the pharmacy, differed significantly (p ***<*** 0.0001), the longest being for alprazolam and the shortest for topiramate. Post hoc tests showed that the difference was significant between each medication (p ***<*** 0.05), except for the comparison between propranolol and primidone (*p* > 0.05) (Fig. 4).

**Table 2:** Pharmaceutical and neurosurgical approaches in Group 6.

	Number (percentage) of patients in Group 6
Pharmaceutical therapy	
Alprazolam	17,911 (44%)
Propranolol	10,410 (25.57%)
Clonazepam	6783 (16.66%)
Antiparkinsonian medication	6129 (15.05%)
Primidone	4521 (11.1%)
Topiramate	138 (0.33%)
Neurosurgical therapy	
Deep brain stimulation	157 (0.39%)
Ablative surgery	33 (0.08%)

We show the number and percentage of patients who picked up their prescription of the listed drugs from the pharmacy at least once during the study period. Most patients tried alprazolam, while propranolol was the second most preferred. Topiramate was dispensed to less than 0.5% of the patients. Less than 0.5% of the patients underwent neurosurgery, and the application of deep brain stimulation was almost 5 times as common as that of ablative surgery.

When the pharmaceutical options were combined, the alprazolam - propranolol therapy was the most common (Table 3).

**Table 3:** The use of drug combinations in Group 6.

Name of the medications	Number (%) of patients taking the combination in Group 6	Time period of medication use (% of the total follow-up time (median and IQR)	Medication use in months (median and IQR)
Propranolol + alprazolam	2749 (6.75%)	35.13 (13.35–68.12)	30.00 (12.00–57.00)
Propranolol + clonazepam	1086 (2.67%)	30.11 (9.34–62.62)	29.45 (9-64.09)
Primidone + alprazolam	962 (2.36%)	32.63 (11.38–60.26)	30.95 (10.18–57.15)
Propranolol + primidone	477 (1.17%)	25.78 (7.49–52.52)	29.00 (7.67-58.00)
Primidone + clonazepam	443 (1.09%)	29.77 (8.99–61.99)	29.00 (10-64.90)
Propranolol + primidone + alprazolam	182 (0.45%)	16.54 (4.66–36.67)	21.90 (5.62–48.42)
Propranolol + primidone + clonazepam	138 (0.34%)	17.38 (5.42–41.69)	22.62 (6.31–55.06)
Topiramate + alprazolam	22 (0.05%)	26.67 (2.87–43.72)	21.97 (3.78–53.32)
Topiramate + clonazepam	20 (0.05%)	20.89 (5.94-62.00)	26.65 (7.10-54.42)
Propranolol + topiramate	19 (0.05%)	22.23 (5.96–48.71)	21.23 (4.46–54.16)
Primidone + topiramate	13 (0.03%)	17.67 (4.48–51.99)	17.90 (4.14–45.73)
Propranolol + primidone + topiramate	4 (0.01%)	8.50 (1.01–20.30)	10.68 (1.33–31.95)

The table presents the number and percentage of patients using the certain drug combination and that how long these patients adhered to it. The most frequently used combination of two medications was propranolol + alprazolam, while propranolol + primidone + alprazolam was the most common triple choice.

Less than 0.5% of the patients underwent neurosurgical procedures such as deep brain stimulation or ablative surgery (Table 2).

In relation to the total number of patients in the Parkinson’s tremor and Not Parkinsonian Tremor Group, less than 4% of patients were later switched from antiparkinsonian therapy to other antitremor therapy, while less than 3% were initially on other anti-tremor drugs that were later changed to antiparkinsonian drugs (Table 4).

**Table 4:** Tendencies for drug switch in Group 4.

The drug switch	Number of patients (% in Group 4)
In the Not Parkinsonian Tremor Group (Group 6)	
Antiparkinsonian drug → propranolol	1107 (2.41%)
Antiparkinsonian drug → primidone	583 (1.27%)
Antiparkinsonian drug → topiramate	22 (0.05%)
In the Parkinson’s disease/syndrome group (Group 7)	
Propranolol → antiparkinsonian drug	863 (1.88%)
Primidone → antiparkinsonian drug	484 (1.05%)
Topiramate → antiparkinsonian drug	12 (0.03%)

We looked for patients in the Not Parkinsonian Tremor Group (Group 6), who used antiparkinsonian medication before switching to one of the antitremor medications. Similarly, in case of the patients in the Parkinson’s disease/syndrome group (Group 7), we searched for those, who were first on an antitremor drug, which was later changed to antiparkinsonian medication. From the evaluation of the treatment switches, a small percentage of patients might be misdiagnosed with Parkinson’s disease/syndrome or essential tremor.

### Prevalence of essential tremor

Our methods identified 37,429 patients with essential tremor (mean age at first tremor diagnosis: 54.93 ± 23.4 years, 44.73% male). Using the population data from the 2011 Census in Hungary^22^, the estimated period prevalence of the disease was 374/100,000. The age-standardized period prevalence of essential tremor for the European Standard Population of 2013^23^ was 388/100,000. If we utilize the results from the Hungarian Census in 2022^24^, the period prevalence of essential tremor was 387/100,000 in Hungary; when standardizing it to the European Standard Population of 2013, it was 378/100,000.

The stratified age and gender analysis in the Probably Essential Tremor Group (Group 8) showed the highest prevalence in the elderly population (1.29% among men and 1.25% among women aged 70–79), the prevalence was similarly high in people over 80 years (0.82% in men and 0.79% among women) and those between 60 and 69 years of age (0.74% for both gender groups). When evaluating by gender, the overall prevalence was 0.35% among men (0.39%) and women (Table 5).

**Table 5:** Stratified age and gender analysis on the prevalence of essential tremor (Group 8) in Hungary.

Age of the patients (years)	Estimated prevalence of ET among men (/100.000 citizens)	Estimated prevalence of ET among women ( /100.000 citizens)
< 20	263	198
20–29	240	182
30–39	123	127
40–49	162	197
50–59	281	287
60–69	737	739
70–79	1292	1253
> 80	823	768
All	351	394

The highest estimated prevalence was found in the elderly population between 70 and 79 years, followed by the prevalence in people over 80 years and those between 60 and 69 years of age. A slight female predominance exists.

When compared to data from other European countries, the estimated prevalence of essential tremor in Hungary is similar to the one assessed in Italy (405/100,000)^16^ but lower than in the Faroe Island^19^, Finland^15^ and Spain^18,25,26^ (Table 6).

**Table 6:** Prevalence of essential tremor in Europe.

Country	Age (years)	Prevalence (/100.000 citizens)	Sample size (number of people)
Faroe Island^19^	≥ 40	3100	1328
Finland^15^	≥ 40	550	3304
**Hungary**	**All ages**	**374**	**59,676**
Italy^16^	All ages	405	7653
Spain^26^	≥ 65	4800	2000
Spain^25^	≥ 65	4800	5278
Spain^18^	> 65	8420	753

In the table, we present published data from other European countries. Our results are similar to the prevalence data from Italy (405/100,000), but the assessed prevalence in the other studies is substantially higher.

## Discussion

In this study, we found that the period prevalence of essential tremor standardized to the European Standard Population was approximately 0.378–0.388% between 2010 and 2020 in Hungary. Alprazolam followed by propranolol were chosen for the longest time period by most of the patients. The propranolol-alprazolam combination was taken in the highest percentage of the medication period by most of the patients. Among neurosurgical procedures deep brain stimulation and ablative surgery were applied in less than 0.5% of the patients, with a possible diagnosis of essential tremor.

Recognition of essential tremor in a broad national health insurance fund database is challenging as standard tests do not support the differential diagnosis processes, which rely on clinical observation^27^. Misdiagnosis is more likely to occur when its severity is subtle^28^. Some studies indicate that the diagnosis of essential tremor is over-applied, with many of the patients having other movement disorders, most commonly Parkinson’s disease or dystonia^28,29^. Tremor is indeed described as a common prodromal presentation of Parkinson’s disease^30^. Physiological tremor is also among the most common alternative diagnoses^31^. Thus, we have attempted to narrow down the population of patients with tremor to essential tremor, based on co-diagnoses and medication use data.

The Hungarian healthcare system has a single health insurance fund managed by the Health Insurance Fund Management Agency (NHIF)^32^. Every Hungarian citizen has a personal identifier^33 ^and all their medical records from hospitals and outpatient services are linked to this identifier and recorded in the database we used.

State health expenditure in relation to GDP in 2021 was 7.4% in Hungary compared to 11% of the EU average^34^. Between 2000 and 2018, 25–30% of the healthcare expenditure was financed by individuals and private companies^35^, but detailed data on each field of the private medical system is scarce. An available survey from Hungary, in which 1100 people were asked, found that among those who sought private medical help, 3% chose neurology^36^. If we extrapolate this finding, excluding private healthcare would introduce only an approximately 1% (30% x 3%) bias in our findings.

In the study period, we initially had 59,676 patients with tremor ICD-10 (International Classification of Diseases-10) codes. Since a short observation time increases the likelihood of misdiagnosis^28^,  we excluded patients with less than two years follow-up period from further analysis. As the next step, we endeavored to screen out patients with Parkinson’s disease, trusting in their need for continuous medication use, which may be initiated after the first years from symptom onset in agreement with the patient^37^. When the patient took antiparkinsonian medication in at least 80% of the total follow-up time, excluding the initial two years, the patient was considered to have Parkinson’s disease/syndrome.

In the remaining Not Parkinsonian Tremor Group, we sorted out patients with alternative causes and co-existing symptoms and tremor-inducing/intensifying medication therapy. Among the conditions, alcohol abuse and hyperthyroidism, among the medications, amiodarone was the most commonly found factor. We saw at least one provoking condition or medication in approximately 9% of the cases. Even though the use of tremor-evoking/enhancing medications does not preclude an underlying essential tremor, we separated this small group of patients to get an estimate that was as accurate as we could ascertain. Finally, we were left with 37,249 people with most probably essential tremor.

There is a marked heterogeneity in prevalence estimates in essential tremor, ranging from 0.01 to 20%, greater than that reported for many other neurological diseases^14^. A systematic review published in 1998 concluded that the prevalence of essential tremor might range from 0.4 to 3.92% across different populations. However, no meta-analysis could be performed on this data because of the disparate methodologies^38^. There are three published meta-analyses on essential tremor prevalence^14,39,40^. In the first one from 2010^39^, the overall prevalence of 28 population-based studies was 0.9% (all ages, limited external validity); in an additional descriptive review of two studies (reported about the patient examination and sensitivity of the methods), the prevalence was 0.4% (all ages). Since more population-based prevalence data became available, the same group conducted another meta-analysis in 2021^14^. Based on 42 studies, they reported a pooled prevalence of 1.33% of the disease (all ages)^14^. A descriptive analytical approach of three studies (reported about the patient examination and sensitivity of the methods) revealed that the median prevalence of essential tremor was 0.4%, and the mean was 0.67% (all ages)^14^. Another meta-analysis from 2021 based on 29 population-based studies reported a prevalence of 0.32% (all ages)^40^, which is comparable to our findings.

Direct comparison of prevalence data in the different meta-analyses is not possible because the proportion of studies conducted in middle-aged (≥ 40 years) or elderly (≥ 60 or ≥ 65 years) populations vary, influencing the results as the prevalence is known to increase with age^40^.

However, the estimated prevalence of essential tremor is highly heterogeneous^14^; it ranges from 0.01%^41^ to 20%^11^, behind which there may be several factors. Even though essential tremor can also be present in the pediatric population^42^, many of the population-based prevalence studies only investigate older people^11,18,21^. However, the age of onset has two peaks: the smaller proportion of young onset cases culminated in their twenties and a more prominent peak given by the sporadic cases starting above 60 years of age^43^. Essential tremor becomes more common with advanced age: the prevalence was indeed shown to increase by 74% for every decade increase in age^14^. The estimated prevalence of essential tremor among the elderly, aged 60 or older, ranges from 0.5%^44^ to 26.1%^45^. A study analyzing a community in Nigeria found a 42.9% prevalence of ET in people aged 85 or older^45^. Males were more likely to be affected than females in many studies^15,17,46^, however the opposite tendency was also reported^47^, other studies found no gender differences in disease distribution^14^. However, the heterogeneity in prevalence data could not be explained by stratifying the data by age and gender^14^.

When interpreting the results from other European countries, it is important to note that most of the data represent small geographical territories of each country^15,16,18,25,26^ and investigate only older people population^18,25,26^,  where the prevalence is known to be higher^40^. Only in Italy^16^ was the study conducted on all age groups, and the results are similar to ours. None of the other studies analyzed therapeutic trends.

Our results regarding the stratification analysis on age are congruent with the literature since the prevalence of essential tremor is higher in the elderly^40^. The prevalence of the disease is generally higher in males^40^, but other studies have also found a slight female predominance^47,48^.

The mild symptoms of essential tremor may not require any treatment, and the pharmacological management of severe essential tremor is limited^8^. Our study found that over one-third of the patients never tried any of the five studied medications (i.e., propranolol, primidone, topiramate, alprazolam, and clonazepam). More than 50% of the patients did not consume these medications in 80% of the follow-up period.

The most commonly used drug was alprazolam, followed by propranolol and clonazepam. Only a small proportion of patients consumed primidone, and less than 0.5% took topiramate.

The effect of the therapies above (with an average improvement of 60%) is limited^7^, which, together with the side effects, the younger age of the patients, and more severe depression, may result in non-adherence^49^. Adherence to any medication is influenced by the effect of the drug on the symptom, its likely unpleasant side effects, and any possible comorbid effects^50,51^. We defined it with two measures in our study. First, with the number of patients to whom the given drug was dispensed at least once. Alprazolam was frequently refilled by the highest percentage of patients, which might be influenced by its anxiolytic effect^52^. A study conducted in the US found that 1–2% of the general population uses alprazolam^52^. This drug was taken by 44% of our Not Parkinsonian Tremor Group (Group 6) patient population, which, might not be attributed to its anti-anxiety effect alone. The second metric of medication adherence we investigated is how many patients refilled a particular drug for the most prolonged period of time. Alprazolam was taken for the longest time by the most patients, followed by propranolol, clonazepam, primidone, and topiramate. In our patient database, the combination of propranolol and alprazolam was the most preferred. The availability of medicines also influences medication habits. In Hungary, benzodiazepine preparations and propranolol can be prescribed by a general practitioner. In contrast, primidone can only be prescribed by a neurologist, and topiramate can be prescribed or authorized for up to one year by a neurologist. Botulinum toxin type A injection has an off-label indication in essential tremor^6^; it is not covered by the national health insurance fund and hence was missing in the therapeutic approaches in our study.

The widespread use of alprazolam might be associated with psychiatric comorbidities and the probably inappropriate prescription of the drug by physicians.

A large retrospective study containing data from more than 120 thousand ET patients from the USA assessed the prevalence of comorbidities among the patients and found that 29% of them had an anxiety disorder, and depression was even more common (44%). They found that in 2019, benzodiazepines were only prescribed to 19% of the essential tremor patients^48^. Even if we extrapolate this finding, the proportion of patients with anxiety would still not fully explain the much higher percentage (44%) of our patients taking alprazolam. Therefore, other factors contribute to it as well. Studies on the rate of the population using benzodiazepines in Hungary have not been conducted yet.

A cross-sectional study from the US analyzing drug utilization among more than 36,000 ET patients found that 17% had at least one prescription for a benzodiazepine^53^. A smaller study of 1,074 ET patients in the US reported that only 2% of the drug prescriptions were for benzodiazepines, specifically clonazepam and diazepam; alprazolam was not studied^54^.

A study investigating the global use of benzodiazepines (namely the defined daily dose (DDD) per 1000 inhabitants per day) found that this metric is more than twice as high in Hungary compared to the average worldwide consumption; however, the study does not contain data on the percentage of the population or the exact number of people taking these drugs^55^.

Regarding the European population, a cross-sectional study conducted in 2019–2020 in seven European countries (Czech Republic, Serbia, Estonia, Bulgaria, Croatia, Turkey, and Spain) found that 14.9% of the investigated older adults (aged 65 or older) were benzodiazepine users, in Croatia, Spain, and Serbia the proportion was more than 30%^56^. In France, benzodiazepines were prescribed to 12.5% of the adult population^57^.

It was revealed in Brazil that primary health care is the most significant source of benzodiazepine prescription, which is frequently inappropriate^58^.

Future research focusing on psychiatric comorbidities among the essential tremor patients in Hungary could explore the cause of high alprazolam consumption in this patient population.

At the time of the study, a small number of patients had switched from antiparkinsonian therapy to other tremor therapy and vice versa. These results also corroborate the diagnostic difficulties in the first period of the disease^28,29^; however, they were rare in our study.

Our data indicated that during this 11-year long time interval, only a small proportion of the essential tremor patients were operated on: 0.39% of the patients received deep brain stimulation, and the number of those who underwent lesioning/ablative surgery was remarkably lower than that. MRI-guided ultrasound thalamotomy was unavailable in the study period and is still unavailable in Hungary. After its introduction in 2016, there have been an increasing number of MRI-guided ultrasound thalamotomies each year, with almost 2000 procedures in 2022 in the United States^59^. ET represented 24% of all DBS indications in the United States between 1993 and 2017, with 1331 implantations per year^60^. According to this published data^60^, 2.7 times more DBS procedures were done for ET in the US than in Hungary per unit population in the time interval overlapping with the time of our study. Economic calculations show that the cost of deep brain stimulation is similar in studies performed in different countries from Europe, Asia and North America and between the distinct indications^61^. Magnetic resonance-guided focused ultrasound thalamotomy has a more favourable cost-effectiveness profile than deep brain stimulation in essential tremor^62–64^. When comparing focused ultrasound thalamotomy and no surgery in people ineligible for neurosurgical procedures with craniotomy, the former is cost-effective due to improved quality-adjusted life years^64^.

In conclusion, Hungary’s estimated prevalence of essential tremor is comparable to the global prevalence found in systematic reviews and meta-analyses. On the other hand, the high use of alprazolam and the small number of neurosurgical interventions require improvements in treatment planning at national level.

Considering the global aging trend, an increase in essential tremor cases can be expected^40^. A broader use of neurosurgical interventions and the possible introduction of focused ultrasound therapy is proposed by conducting cost-effectiveness studies and, in parallel, by establishing national therapeutic guidelines and providing education to physicians.

## Limitations

The prevalence of essential tremor may be underestimated as many patients do not consult a doctor with mild symptoms and do not take medication^14^.

The prevalence determined was a rough estimate from the National Health Insurance Fund database based on physicians’ coding of tremor diagnoses. Given the large number of patients, reviewing individual medical records was impossible. Nevertheless, we tried to refine it by a matched, parallel analysis of medication use data. We did not consider data from private care because they did not have mandatory reporting before June 01, 2020.

Our database is anonymized, with no personal identifiers. Therefore, we were unable to validate a subset of cases from it. Consequently, we could not determine the exact values for the sensitivity and specificity of our methodology. A few factors could influence sensitivity and specificity, such as the reliance on ICD-codes and medication use and excluding private healthcare (approximately 1% bias).

Analyzing regional or urban-rural differences in prevalence was impossible because the database does not contain information regarding patients’ places of residence. Nevertheless, addressing this question in the future can further characterize the prevalence of essential tremor.

## Methods

### Description of databases and identification of tremor patients

In order to estimate the prevalence of essential tremor in Hungary, we used two independent databases: the databases of the National Health Insurance Fund covering patient care from all hospitals and specialist outpatient services (between 2004 and 2020); and the pharmacy database of medication refills from all pharmacies throughout the country (between 2010 and 2020). The original patient identifier codes were anonymized and we used encrypted identifiers. The study was performed according to the Declaration of Helsinki. All personal data protection regulations were followed. The study was approved by the Medical Research Council of Hungary (ETT TUKEB IV/7136- 1 /2021/EKU). Due to the retrospective nature of the study, the Medical Research Council of Hungary waived the need of obtaining informed consent.

Each patient had a constant encrypted identifier in the whole study period in the two distinct databases, allowing us to link the matching records. As pharmacy medication refills data were available only from 2010 on, we only analyzed the overlapping 11-year-interval between 2010 and 2020.

In the first step we analyzed the inpatient and specialist outpatient databases and selected those who, during these years, received at least once a primary or secondary diagnosis of tremor (Group 2). We considered the reported tremor diagnoses if they were established by neurologists and other specialists. With this selection criterion, we included patients who might have only consulted with their general physician to reduce the possible underrepresentation of mild essential tremor cases. For the diagnosis codes we used the 10th Edition of the International Classification of Diseases (ICD-10) and searched for the ICD-codes accordingly: G2500 (essential tremor), G2510 (drug-induced tremor), G2520 (other specified forms of tremor), R2510 (tremor, unspecified).

As other symptoms of Parkinson’s disease are likely to appear within two years of the onset of monosymptomatic tremor in Parkinson’s disease^65^, we restricted the study sample to patients with a documented follow-up period of at least 2 years during the study period (Group 4) to increase the robustness of the diagnosis. If patients died or our study period had come to an end within two years after receiving the first tremor diagnosis, we excluded that data from further analysis (Fig. 1).

### Grouping the multiple causes of tremor

As the next step, we sorted out patients with Parkinson’s disease (Group 7) using the following criteria: the pharmacy records had to indicate that the patient used antiparkinsonian medication in at least 80% of the follow-up time (Anatomical Therapeutic Chemical Classification System - ATC - code: N04). When calculating this ratio, we subtracted two years from the actual follow-up time of the patient because of the uncertainty of the diagnosis of Parkinson’s disease, which could lead to alternative treatment strategies. After removing the Parkinson’s patients, the remaining group of patients (Not Parkinsonian Tremor Group: Group 6) was further investigated (Fig. 1).

In addition to Parkinson’s disease, other diseases or signs might also receive a tremor diagnosis code in the medical records. In Group 6, we searched for the following ICD-10 codes for alternative diagnoses in tremor syndromes: multiple sclerosis (G35H0, G3680, G3690, G3710, G3730, G3780, G3790), hyperthyroidism (E0510, E0530, E0600, E0610, E0620, E0640, E0650, E0690, E0780, E0790), alcohol abuse (F1010, F1020, F1030, F1040, R7800, T5180, T5190, Y9090, Y9100, Y9110, Y9120, Y9130, Y9190, Z7210) and Wilson’s disease (E8300) as well as for additional diagnosis or signs that could be associated with tremor: dystonia (G2400, G2410, G2420, G2440, G2480, G2490), torticollis (G2430) and blepharospasm (G2450).

We also examined the refills of the following drugs, which may evoke enhanced physiological tremor: amiodarone, valproic acid, l-thyroxin and lithium.

We have performed stratification analysis by gender and age in the Most Probably Essential Tremor Group (Group 8, Table 5).

### Therapeutic approaches

In the Not Parkinsonian Tremor Group (Group 6) we examined the pharmaceutical therapies from a number of different perspectives. Within the pharmacy database, we evaluated the use of the most frequently applied medications: propranolol, primidone, and topiramate for essential tremor, antiparkinsonian medications (ATC N04), and two benzodiazepines (alprazolam and clonazepam). For each drug, we collected the following information: the time of the first and last acquisition of medication during the study period, the interval between them, and the number of medication packages dispensed to the patient.

We determined the number of patients to whom none of the selected medicines were dispensed for at least 20% of the study period.

If the patients took medication, we observed which drug they preferred for the most prolonged time period.

We then calculated the temporal ratio of each medication use individually, dividing the time interval of medication refills by the total follow-up time. We further investigated the drug combinations, the number of patients utilizing the combinations, the length of their utilization time period and the temporal ratio of their use were assessed.

Furthermore, we looked into changes in therapy: we assessed the number of patients in Group 6 who first was on antiparkinsonian medications (ATC N04) and later switched to one of the antitremor drugs (propranolol, primidone, and topiramate, alprazolam and clonazepam). Among the patients who were finally considered to have Parkinson’s disease/syndrome (Group 7), we selected those who were first on an antitremor medication, and it was later changed to antiparkinsonian therapy.

In addition to medications, we searched the database for surgical procedures, such as ablative surgery and deep brain stimulation.

### Statistical analysis

The estimation of the prevalence of essential tremor in Hungary was based on the Hungarian population data. We calculated the crude period prevalence in Hungary using data from the Central Bureau for Statistics (Census 2011^22^ and Census 2022^24^). With the help of the direct standardization method, our results were age-adjusted to the European Standard Population of 2013. We also compared the results with existing prevalence data from other European countries. For data analysis, we used descriptive statistics. Normality was evaluated with Shapiro–Wilk test. We used OriginLab software (OriginLab Corporation, Northampton, MA, USA) for statistical analysis and data presentation. Krusall-Wallis test (one-way ANOVA on ranks) was used with the post-hoc Dunn’s test to detect possible differences between the durations of medication use.

## Data Availability

The dataset for this study can be requested from the corresponding author by qualified researchers, in accordance with the limitations set by the ethical approval.
